# Sophisticated viral quasispecies with a genotype-related pattern of mutations in the hepatitis B X gene of HBeAg-ve chronically infected patients

**DOI:** 10.1038/s41598-021-83762-4

**Published:** 2021-02-18

**Authors:** Maria Francesca Cortese, Carolina González, Josep Gregori, Rosario Casillas, Luca Carioti, Mercedes Guerrero-Murillo, Mar Riveiro-Barciela, Cristina Godoy, Sara Sopena, Marçal Yll, Josep Quer, Ariadna Rando, Rosa Lopez-Martinez, Beatriz Pacín Ruiz, Selene García-García, Rafael Esteban-Mur, David Tabernero, Maria Buti, Francisco Rodríguez-Frías

**Affiliations:** 1 Liver Unit, Liver Disease Laboratory-Viral Hepatitis, Vall d’Hebron Research Institute, Passeig Vall d’Hebrón, 119-129 Barcelona, Spain; 2 Liver Pathology Unit, Departments of Biochemistry and Microbiology, Vall d’Hebron University Hospital and Universitat Autònoma de Barcelona, Barcelona, Spain; 3 Roche Diagnostics SL, Sant Cugat del Vallès, Spain; 4 Department of Experimental Medicine, University of Rome Tor Vergata, Rome, Italy; 5 Department of Microbiology, Vall d’Hebron University Hospital, Barcelona, Spain; 6 Centro de Investigación Biomédica en Red de Enfermedades Hepáticas y Digestivas (CIBERehd), Instituto De Salud Carlos III, Madrid, Spain; 7 Liver Unit, Department of Internal Medicine, Vall d’Hebron University Hospital and Universitat Autònoma de Barcelona, Barcelona, Spain

**Keywords:** Infectious diseases, Haplotypes, Mutation, Sequencing

## Abstract

Patients with HBeAg-negative chronic infection (CI) have not been extensively studied because of low viremia. The HBx protein, encoded by *HBX*, has a key role in viral replication. Here, we analyzed the viral quasispecies at the 5′ end of *HBX* in CI patients and compared it with that of patients in other clinical stages. Fifty-eight HBeAg-negative patients were included: 16 CI, 19 chronic hepatitis B, 16 hepatocellular carcinoma and 6 liver cirrhosis. Quasispecies complexity and conservation were determined in the region between nucleotides 1255 and 1611. Amino acid changes detected were tested in vitro. CI patients showed higher complexity in terms of mutation frequency and nucleotide diversity and higher quasispecies conservation (*p* < 0.05). A genotype D-specific pattern of mutations (A12S/P33S/P46S/T36D-G) was identified in CI (median frequency, 81.7%), which determined a reduction in HBV DNA release of up to 1.5 log in vitro. CI patients showed a more complex and conserved viral quasispecies than the other groups. The genotype-specific pattern of mutations could partially explain the low viremia observed in these patients.

## Introduction

Currently, around 257 million people worldwide are chronically infected by hepatitis B virus (HBV), and HBV infection remains the main cause of death due to viral hepatitis (WHO report, July 2018). HBV has a small (3.2 kb), partially double-stranded DNA genome with 4 highly overlapping open reading frames (ORFs): the polymerase, surface, Core, and X genes (*HBX*)^1^. HBV has been classified into 8 major genotypes (A–H) and several sub-genotypes. Genome replication requires a reverse transcription step, which lacks proofreading control, so the HBV genome acquires spontaneous mutations, reaching a mutation rate resembling that of RNA viruses^2^. As a result of this variability, HBV circulates as a quasispecies (QS), a complex mixture of closely related viral genetic variants referred to as haplotypes.

The HBx protein, encoded by the *HBX* gene, has a key role in HBV infection and disease progression. It is an essential element to initiate a productive infection^3,4^, enables expression of covalently closed circular DNA (cccDNA) by acting on its epigenetic control^3,5^, and interacts with cellular proteins, such as DDB1 and cyclic-AMP-response element binding protein (CREB), involved in HBV replication^3,6^. Furthermore, HBx interferes with other cellular pathways, thus making it a multifunctional and pleiotropic protein with a major role in the development of hepatocellular carcinoma (HCC)^7^.

HBV patients are clinically classified based on determination of several viral and biochemical markers (eg, HBeAg, viremia, alanine aminotransferase ALT] level)^8^. Those with HBeAg-negative (HBeAg-ve) chronic HBV infection (CI), previously known as inactive carriers, constitute a unique clinical group characterized by viremia ≤ 2000 IU/mL, low HBsAg levels^8^, normal ALT concentrations, and minimal or absent hepatic necroinflammation. These patients have a favorable prognosis, but the risk of HCC and hepatitis reactivation after immunosuppressive therapy persists, and continuous periodic follow-up testing is advisable^9,10^.

By studying these patients, valuable information about the mechanisms that enable control of viral replication could emerge, suggesting potential targets for new therapeutic approaches. However, because of their low HBV viral load, the HBV genome has not been extensively studied in this patient group and, to date, the mechanism behind their low replication rate is currently unknown. Although the immune response is likely an important element in controlling HBV replication, no great differences in CHB-specific T immunity or cytokine/chemokine levels have been reported between CI and chronic hepatitis B patients^11–13^. Could the variability and complexity of the viral genome be determinant factors in this clinical stage? To explore this question, we analyzed samples from a group of well-characterized HBeAg-ve chronically infected patients, and compared the results with those of the chronic hepatitis groups, including patients with liver cirrhosis and HCC. In previous studies, the presence of insertions and deletions in the 3′ end of *HBX* has been related to variations in disease progression^14,15^. We deeply analyzed this region by next-generation sequencing (NGS) and further confirmed this variability (manuscript in preparation); hence we focused our study on a region that includes the 5′ end of the *HBX* gene and the upstream non-coding region. In a previous study, we observed some hyper-conserved regions in this portion of *HBX*^16^, but we did not examine the conservation profile related to different clinical groups at that time.

The aim of this study was to identify genetic factors in the 5′ *HBX* quasispecies that characterize CI patients, in order to elucidate their relationship with the control mechanisms affecting viral replication in this population.

## Results

### Chronic infection: An inactive state of infection with higher QS functional complexity

The region of interest was amplified by a previously reported 3-round PCR protocol^16^. The external PCR did not affect the composition of the HBV quasispecies, as was confirmed by comparing the QS complexity indices obtained by analyzing serial dilutions of two samples amplified with 3- or 2-round PCRs (Supplementary Fig. S1, *p* > 0.05).

Only patients with ≥ 5000 reads (sequencing data) were included in the analysis, resulting in 52/57 patients in total: 15/16 CI, 17/19 CHB, 5/6 LC, and 15/16 HCC. Of note, in 7 of the 15 HCC patients the tumor had developed in the presence of cirrhosis, and in 3 patients this information was not available. Demographic, virologic, and serologic characteristics are reported in Table 1.

**Table 1:** Main viral and serologic characteristics of the clinical groups enrolled in the study.

	CI (n = 15)	CHB (n = 17)	HCC (n = 15)	LC (n = 5)	*p*
Age, median [Q1–Q3]	55 [37–62]	43 [35–55]	62 [55–67]	50 [42–59]	**0.016**
Sex, % men (n)	53.3% (n = 8)	64.7% (n = 11)	93.3% (n = 14)	100% (n = 5)	** < 0.0001**
**Genotype,**^**a**^**%**					
A	2.7	18.4	47	1.7	**0.004**
C	13.8	8	13.8	15.3	0.88
D	73.7	38.5	38.7	76.7	0.02*
E	0.1	16.4	0	0.1	0.09
F	9.3	5.9	0	6.2	0.639
H	0.5	12.9	0.6	0	0.2
HBV DNA, logIU/mL, Median [Q1–Q3]	3.1 [2.4–3.4]	4.8 [4.0–5.5]	5.6 [4.9–6.0]	4.7 [3.8–6.4]	** < 0.0001**
ALT, IU/mL median[Q1–Q3]	28.0 [18.5–31.5]	47.5 [38–84.5]	61 [48.5–79.45]	49 [42–75.7]	**0.004**
Platelets × 10^9^/L, median [Q1–Q3]	237 [219–289]	220 [166–240]	140.5 [101.2–167.5]	112 [98–134]	**0.0004**

*CHB* chronic hepatitis B, *LC* liver cirrhosis, *HCC* hepatocellular carcinoma, *CI* chronic infection; Q1, 25th percentile; Q3, 75th percentile; *HBV* hepatitis B virus, *ALT* alanine aminotransferase. *p* values (*p*) were obtained by applying the Kruskal–Wallis test in median age, HBV DNA and ALT, and by ANOVA in genotype distribution. Statistically relevant *p* values are reported in bold.

*p > 0.05 when Tukey multiple comparison was implemented.

^a^Genotype was evaluated by NGS sequencing, as described^16^.

After applying quality filters, 1,310,514 sequences were obtained, which yielded a median [Q1–Q3] of 20,821 [16,149–29,848] sequences per patient. NGS data were submitted to the GenBank SRA database (BioProject accession number PRJNA437055, BioSample accession numbers in Supplementary Table S1).

In the comparison of viral quasispecies complexity, no differences were found between CI patients and the other groups for Shannon entropy (Sn), (median 2.26, 2.03, 1.72, and 2.94 for, respectively, CI, CHB, HCC, and LC) (Table 2). Similar findings were observed for the Gini–Simpson index (G) with a median of 0.81 for CI, 0.77 for CHB, 0.73 for HCC, and 0.89 for LC (Table 2).

**Table 2:** Viral quasispecies complexity in the different clinical groups.

	Sn Median [Q1–Q3]	G Median [Q1–Q3]	Mf Median [Q1–Q3]	π Median [Q1–Q3]
CI	2.26 [1.75–2.62]	0.81 [0.76–0.86]	0.017 [0.009–0.036]	0.027 [0.017–0.043]
CHB	2.03 [1.35–2.37]	0.77 [0.59–0.83]	0.004 [0.003–0.017]	0.007 [0.005–0.023]
HCC	1.72 [0.92–2.13]	0.73 [0.37–0.79]	0.004 [0.002–0.008]	0.005 [0.003–0.013]
LC	2.94 [2.39–3.17]	0.89 [0.81–0.92]	0.030 [0.012–0.052]	0.040 [0.02–0.056]

*CHB* chronic hepatitis B, *LC* liver cirrhosis, *HCC* hepatocellular carcinoma, *CI* chronic infection, *Q1* 25th percentile, *Q3* 75th percentile, *HBV* hepatitis B virus, *ALT* alanine aminotransferase. *p* values (*p*) were obtained by applying the Kruskal–Wallis test in median age, HBV DNA and ALT and by ANOVA in genotype distribution. Statistically relevant *p* values are reported in bold.

*p* > 0.05 when Tukey multiple comparison was implemented.

Genotype was evaluated by NGS sequencing, as described^17^.

**Figure 1:**
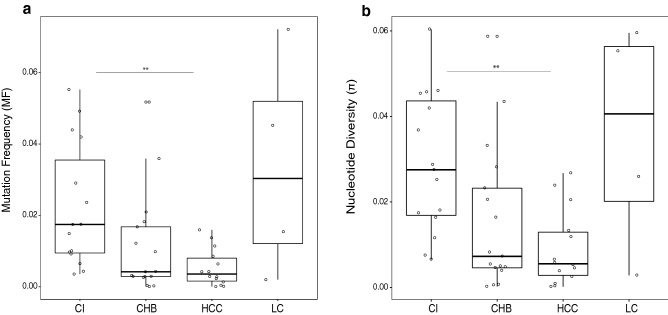
Mutation frequency (Mf) and population nucleotide diversity (π) comparisons. Boxplots show comparisons of Mf (**a**) and π (**b**) values between the 4 clinical groups. Each dot represents a single patient. Asterisks above the boxes denote specific *p* values (2 asterisks < 0.01) obtained by performing the Kruskal–Wallis test plus Dunn posthoc test and adjusted with the Bonferroni correction. *CHB* chronic hepatitis B; *CI* chronic infection, *HCC* hepatocellular carcinoma, *LC* liver cirrhosis.

By contrast, in the functional parameters, the CI group showed, respectively, 4.2- and 4.9-fold higher mutation frequency (Mf) values than CHB and HCC (median in CI 0.017, CHB 0.004, and HCC 0.004, *p* value related to HCC 0.005) (Table 2; Fig. 1a). LC showed the highest mutation frequency (median 0.030), but as few LC patients were included in this study, these results should be confirmed in a larger cohort. Consistent findings were recorded for the nucleotide diversity index (π), in which CI showed a value up to fivefold higher than the CHB or HCC groups (median 0.027, 0.007, and 0.005 respectively, for CI, CHB and HCC (*p* = 0.005) (Table 2; Fig. 1,b). Again, LC showed the highest nucleotide diversity value (median = 0.04).

### Similar quasispecies conservation between patient groups: Information content study

By calculating the information content and applying sliding window analysis, we confirmed the presence of highly conserved nt and aa regions previously reported by our group^16^ (Supplementary Fig. S2 and S3).

**Figure 2:**
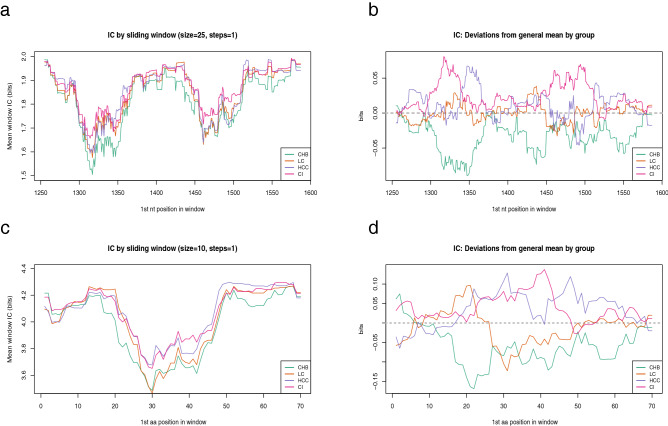
Intergroup variability of the information content. (**a**) The sliding window analysis is the result of the mean information content (bits) of the 25-nt or 10-aa long windows with a displacement between them of 1-nt or 1-aa obtained by multiple alignments of all the QS haplotypes. A. Sliding window analysis of nt sequences in the various clinical groups. Each line displays a specific clinical group (CHB in green, LC in orange, HCC in purple, and CI in pink). (**b**) Representation of the intergroup variability in nt conservation. Each line indicates a group of patients. The information content general mean is highlighted with a grey dotted line (positive deviation indicates greater conservation, whereas negative deviation indicates lesser conservation). (**c**) Sliding window analysis of aa sequences in the various clinical groups. (**d**) Intergroup variability in aa-conservation. Grey dotted line indicates the general mean of information content.

Conservation at both the nt and aa level was similar, but not identical, in all groups (Fig. 2a, c). By comparing the information content standard deviation of each group relative to the overall mean we observed that CI and LC sequences were the most highly conserved, mainly in the non-coding portion encompassing nt 1300–1375 (*p* = 0.005 between CI and CHB information content deviation) (Fig. 2b). The aa comparison yielded similar results: CI showed higher conservation than CHB, especially in the region between aa 20 and 50 (*p* = 0.019) (Fig. 2d).

### Genotype-specific pattern of mutations

To determine whether the presence of certain aa mutations might enable differentiation between the clinical groups, all haplotypes were aligned against each specific genotype consensus; some genotype-specific changes were observed (Supplementary Fig. S4). Inspection of the overlapping polymerase ORF showed some mutations related to the genotype consensus, but there were no differences between the clinical groups.

**Figure 3:**
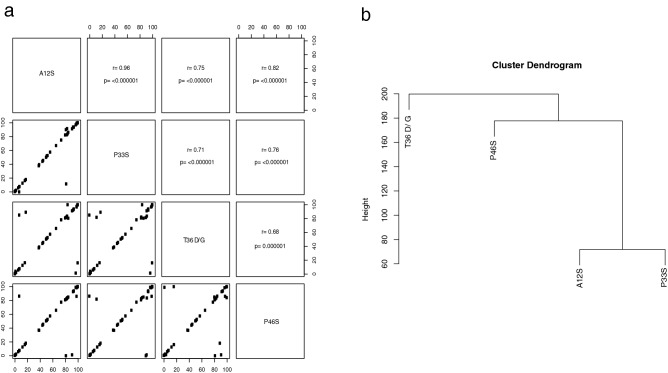
Pattern of mutations in genotype D haplotypes. (**a**) Spearman correlation between the frequencies of the amino acid changes highlighted in genotype D haplotypes. Each dot represents the change’s frequency in each patient. Rho and *p* values are reported. (**b**) Hierarchical cluster dendrogram of the observed mutations. The y-axis is a measure of closeness of an individual mutation or a cluster.

None of the mutations in haplotypes from genotypes A, C, E, F, or H were differentially represented between the four clinical stages. However, in the case of genotype D, the A12S, P33S, P46S, and T36A/D/G mutations were more highly represented in one specific group, CI patients. These mutations correlated with each other (rho ≥ 0.68, *p* ≤ 0.00001) (Fig. 3a), thereby forming a pattern of mutations (Fig. 3b).

**Figure 4:**
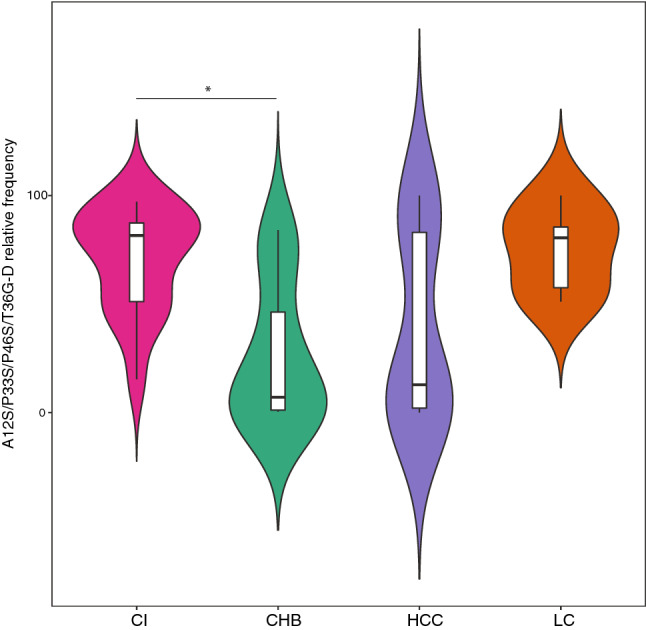
Frequency of the genotype D-specific mutational pattern. The violin plot compares the frequency of the mutational pattern (A12S/P33S/P46S/T36G-D) between the clinical groups. The internal white bar represents the interquartile range with the median in a black horizontal line, whereas the vertical line shows the 95% confidential interval. The violin shape indicates the probability density, in which wider sections indicate a higher probability that a patient will have a given frequency of the mutational pattern. The *p* value was determined by performing the Kruskal–Wallis test plus and the posthoc Dunn test, and is reported as asterisks (1 asterisk, < 0.05).

This pattern was found in CI patients at a rate around 12-fold higher than that of the CHB group (median [Q1, Q3] of 81.6 [51.1–87.4] in CI vs CHB 7.1 [1.2–46.3], *p* = 0.026) (Fig. 4). A similar high rate was observed in LC (median [Q1; Q3] of 80.5 [57.5; 85.5]), however, due to the limited number of patients in this group, the difference was not statistically significant. On analysis of the total of patients, no correlations were found between viral load and frequency of the mutation pattern, whereas a weak correlation was observed when only CHB and IC patients were analyzed (*p* = 0.02 and rho =  −0.4). Presence of the mutation pattern did not extremely modify the tridimensional structure of the HBx protein relative to the wild type (wt) (Supplementary Fig. S5). However, analysis of the effects of mutations on HBx stability (ΔΔg) between the wt protein and both patterns showed a reduction in protein stability (ΔΔg < 0). Of note, the A12S/P33S/P46S/T36D pattern presented a ΔΔg lower than the pattern with T36G (ΔΔg of − 1.4 and − 1.85 for respectively, T36D and T36G), indicating greater instability.

### Lower in vitro HBV expression in the presence of the mutation pattern

**Figure 5:**
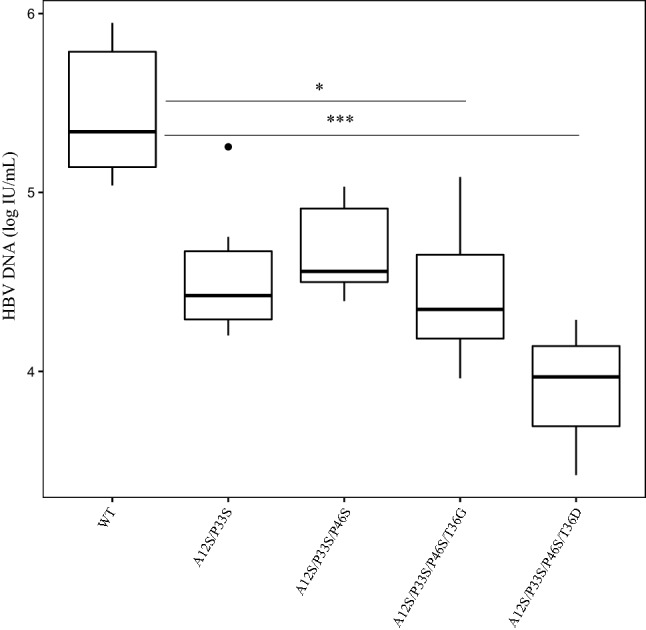
In vitro HBV expression in the presence of HBx mutations. The boxplot represents HBV particle release (HBV DNA in log IU/mL) in cell supernatants at 5dpt in the presence of wild type (wt) or mutated HBV monomers. The Kruskal–Wallis plus Dunn test was implemented and the *p* values obtained are reported as asterisks, where the number of asterisks identifies a specific *p* value interval (1 asterisk, < 0.05 and 3, < 0.0001). Results are the median of three experiments performed in duplicate.

To investigate the effects of HBx aa changes on HBV expression, mutations were tested in vitro following the order of hierarchical clustering. All mutations were found to reduce viral particle release at 5 days post-transfection in cell supernatants relative to wt HBV (Fig. 5).

Specifically, HBV DNA showed a > 14% decrease in the presence of the double mutant A12S/P33S and the triple mutant A12S/P33S/P46S (median [Q1–Q3] 4.4 [4.3–4.7] logIU/mL, and 4.6 [4.5–4.9] logIU/mL, respectively, versus 5.3 [5.1–5.8] for wt HBV). Furthermore, a reduction of 18.5% (4.3 [4.1–4.6] logIU/mL) occurred in the presence of the quadruple mutant A12S/P33S/P46S/T36G (*p* = 0.047). Overall, the decrease in HBV viremia was between 0.8 and 1 log. Finally, in the presence of the quadruple mutant A12S/P33S/P46S/T36D, there was a 25.6% reduction in HBV DNA (4[3.7–4.1] logIU/mL), which accounted for a 1.4-log drop in HBV viremia relative to the wt virus (*p* = 0.00012). This trend was also observed at the protein level, where the quadruple mutant determined a reduction in HBcrAg release of 1.5 logU/mL related to wt (Supplementary Fig. S6).

## Discussion

Because of the low viral load in chronically infected HBeAg-ve patients, it has been difficult to analyze the HBV genome in this population. Therefore, little is known about the virological basis of their specific clinical characteristics. To investigate the viral QS in HBeAg-ve chronic infection, samples from 16 CI patients were analyzed by NGS, and the results obtained were compared with those of chronic hepatitis patients in different clinical stages.

The QS was more complex in CI than in chronic hepatitis patients at the functional level. In agreement with these findings, high complexity and spontaneous reverse transcriptase mutations (in the A-B interdomain, overlapping with the HBsAg *a* determinant) have been reported in the viral QS related to the immune-tolerant and immune-active states^18^. Furthermore, an increase in haplotype number in a region including the 5′-end of *HBX* was detected in an HBeAg-positive woman (previously defined as immune-tolerant) who seroconverted to HBeAg-negative (CI state)^19^. Our results suggest that the accumulation of nt mutations in the QS of CI patients could influence HBV replication, thus providing a possible explanation for the low viral replication rate in this population.

Clinical groups such as CHB and HCC patients are generally found to have highly replicative and/or carcinogenic variants, but curiously, the QS composition was less complex in these patients, suggesting that certain variants had been selected to guarantee HBV replication and persistence. We found the most complex QS in the LC group, but as very few patients with cirrhosis are attended in our outpatient clinic, only a small number were included in the study, which could make these results less reliable.

The portion of the *HBX* gene examined here comprises the 5′ end and the upstream non-coding region, where some hyper-conserved regions have been reported by our group. The current analysis of QS conservation in different, well-characterized, clinical groups confirms these previous results and supports the idea that these regions could be useful targets for gene therapies, as they would be effective regardless of the clinical and virological conditions.

In light of the higher functional QS complexity observed in CI patients, and taking into account that the nt and aa conservation findings were similar but not identical between the different clinical groups, we examined the intergroup conservation variability. Surprisingly, sequences from CI patients were the most conserved at both the nt and aa level, particularly relative to the CHB group. The difference between CI and CHB conservation was higher in the upstream non-coding region of *HBX* (nt 1250–1350, where several *HBX* transcript initiation sites have been reported^20^) and in the protein dimerization site (aa 20–50)^21^. The lower conservation of CHB haplotypes may suggest that nt and aa variability help HBV to re-adapt to the external environment and guarantee replication. Conversely, in the CI QS, higher conservation may indicate selection of more highly conserved and probably less replicative haplotypes, which could presumably promote viral persistence.

These results seem to indicate a more complicated HBV QS in CI patients. The high functional complexity would be due to the presence of a large number of mutated haplotypes at a low frequency, which do not, however, affect the nt and aa conservation of the main population. These results should be confirmed in a larger sample that includes study of the intracellular viral quasispecies, mainly in patients with HCC where compartmentalization of the viral variants between tumor and adjacent non-tumor tissue has been observed^22^.

To explain the limited viral replication in CI patients, we investigated the presence of aa changes. Determination of aa mutations in these patients could be helpful to better classify those falling into the “grey zone” (viremia 2000–20,000 IU/mL and/or marginally elevated ALT) whose management is still difficult due to the lack of factors that distinguish this intermediate state from chronic HBeAg-ve infection or hepatitis^23^. *HBX* and its encoded protein have a key role in HBV replication and disease progression, and may be determinant for the low replication activity seen in CI. Recently, a higher *HBX* mutation rate was reported in CI patients than in “active” chronic hepatitis patients^24^. Nonetheless, the study investigated only dominant mutations determined by Sanger sequencing (at a minimum frequency of 15–20%)^25^. Deletions in the *HBX* 3′-terminal end were detected by NGS in the above-mentioned woman who experienced HBeAg seroconversion^19^. In the present study, the presence of mutations was analyzed by aligning haplotype sequences obtained by NGS with their corresponding genotype consensus. As genotyping was performed considering haplotype sequences, we were able to detect subtle mixtures of genotypes, usually not identified by Sanger sequencing, in keeping with another NGS study, where mixed genotypes were reported in the HBV X and precore region^26^. Notably, we observed a genotype-specific viral evolution. The HBV QS may evolve differently depending on the genotype, and thereby, differentially influence disease progression and therapy outcome^27,28^. Specific mutations in the HBsAg C-terminal domain of genotype D HBV have been associated with viremia < 2000 IU/mL^29^, and certain HBx mutations highly associated with HCC have been reported specifically in genotypes C and D^30,31^. Here, we detected a pattern of aa changes (A12S/P33S/P46S/T36G-D) that was highly represented in low-replicative groups, mainly genotype D CI haplotypes. These observations illustrate the importance of using NGS to accurately identify viral genotypes, useful information for following up the disease.

The frequency of the mutation pattern showed a weak inverse correlation with HBV viremia in CI and CHB patients, suggesting a relationship between viral expression and mutations. This correlation was lost when HCC and LC patients were included in the analysis. This observation suggests that other mechanisms (eg, mutations in other genes) may influence HBV expression in these last two groups. The mutation pattern identified partially involved the HBx Ser/Pro-rich dimerization site^21^, and was mainly characterized by replacement of a hydrophobic aa (alanine or proline) by a polar aa (serine). This could be relevant, as some highly conserved polar aa (eg, Ser25 and Ser41) within the Ser/Pro-rich domain are targets of post-translational changes^32^. The potential addition of new phosphorylation and O-β-glycosylation sites could interfere with the tridimensional structure of the HBx protein and thereby limit its trans-activating activity.

In vitro investigation of HBV expression in the presence of the A12S/P33S/P46S/T36G pattern showed an approximately 1 log reduction in HBV expression. More marked inhibition was detected in relation to A12S/P33S/P46S/T36D. Analysis of protein stability showed that HBx was less stable in the presence of both patterns, more evidently so in relation to the T36D pattern, indicating that the specific threonine to aspartate change may further affect HBx stability and consequently, its trans-activating activity.

The results of this study provide insight into the composition of the HBV QS in CI patients. However, further work is required to characterize the mechanisms responsible for the complex viral population observed and to determine the role of highly mutated haplotypes in viral replication. A genotype-specific pattern of mutations that reduced viral replication was detected in the CI QS. Application of ANOVA plus the Turkey test on genotype distribution among the haplotypes showed no difference in the prevalence of genotype D between the clinical groups. Nonetheless, genotype D was highly predominant in CI patients compared to the others and this may have affected the prevalence of the mutation pattern in this group. Studies in larger samples with various genotypes are needed to confirm the association between mutations and CI status and to detect other genotype-specific aa changes that may enable differentiation between HBV clinical groups. Additional *in vitr*o and in silico studies are required to further understand whether and how these mutations interfere with the activity and tridimensional structure of HBx.

In summary, the HBV viral population at the *HBX* 5′ end was investigated by NGS analysis in a group of HBeAg-ve chronically infected patients. The sophisticated (both conserved and complex) QS characterized may explain the limited viral replication in this patient population. The presence of aa mutations specific to a certain genotype underscores the need to accurately genotype the HBV virus during follow-up. The pattern of mutations observed in this study could help to better classify chronically infected HBeAg-ve patients and the state of low viral replication rate.

## Material and methods

### Patients and samples

All experiments and methods were performed in accordance with relevant guidelines and regulations. The study was approved by the Ethics Committee of Vall d’Hebron Research Institute (PR(AG)411/2016 and PR(AG)146/2020). Patients were enrolled from the population attending the outpatient clinics of Vall d’Hebron Hospital (Barcelona, Spain). All patients were informed about the aims of the project, and signed an informed consent form.

Only those samples with a viral load > 100 IU/mL were included. Patients were stratified according to European guidelines. Briefly, chronic infected patients (CI) presented a viremia ≤ 2000 IU/mL or normal ALT (≤ 40 IU/mL). In case of viremia < 20,000 IU/mL with normal ALT (grey zone), patients were followed-up, and only those with persistent low viremia and no signs of liver damage were classified as CI patients. Chronic hepatitis patients (CHB) presented a viremia > 2000 IU/mL with ALT > 40. A plasma sample was collected from 16 CI, 19 CHB, 16 patients with signs of hepatocellular carcinoma (HCC), and 6 with liver cirrhosis (LC). Patients were all HBV-monoinfected and HBeAg-ve. Demographic, virologic, and serologic characteristics are reported in Table 2.

HBsAg and HBeAg were tested using commercial enzyme immunoassays (COBAS 8000 analyzer, Roche Diagnostics). HBV DNA was quantified by real-time PCR (COBAS 6800, Roche Diagnostics) with a detection limit of 10 IU/mL.

### *HBX* amplification and sequencing

The region of interest encompassed nt 1255–1611, which includes the 5′ end of the *HBX* coding region (nt 1374–1611, corresponding to aa 1–76 in the coded protein) and the upstream non-coding region.

HBV DNA was extracted from 500 µL of plasma with the QIAamp UltraSens Virus Kit (QIAGEN) according to the manufacturer’s instructions. The region under study was amplified using a 3-round nested PCR protocol that enabled amplification of samples with viremia > 100 IU/mL. Briefly, the first-round PCR was performed using external primers (forward 5′-TGTATTCCCATCCCATCATC at position nt 599 and reverse 5′-AGWAGCTCCAAATTCTTTATAAGG, at nt position 1936) with the following protocol: 95ºC for 5 min, followed by 35 cycles of 95ºC for 20 s, 53ºC for 20 s, 72ºC for 15 s, and finally, 72ºC for 3 min. The volume of extracted DNA added to the amplification mix (5–10 μL) differed depending on the initial viral load of the sample, and the amount of water used was proportionately adjusted to reach the same final total volume.

The second-round PCR (using primers carrying M13 universal adaptor) and third-round PCR (using primers including a unique multiplex identifier sequence [MID] for each sample/patient) were performed as described^16^, To ensure that the 3-round PCR did not change the viral populations, a control run with a 2-round PCR (excluding the external PCR) was performed in 2 CHB samples serially diluted up to e+03 IU/mL. All PCR steps were performed using high-fidelity Pfu Ultra II DNA polymerase (Stratagene, Agilent Technologies). PCR products were purified using the QIAquick Gel Extraction Kit (QIAGEN) following the manufacturer’s instructions, and DNA quality was evaluated using the Agilent 2200 TapeStation (Agilent Technologies). Purified PCR products were quantified using the Quant-iT PicoGreen dsDNA Assay Kit (Life Technologies) to equilibrate representation of each sample in the pool, and then sequenced by NGS on the Illumina MiSeq platform (Illumina Inc., San Diego, CA, USA).

Sequences obtained were bioinformatically filtered as previously described^33^, which resulted in unique sequences covering the full amplicon (haplotypes) that form the viral QS. Haplotypes included in the next analyses had common reverse and forward sequences, and an abundance of ≥ 0.1% in the QS complexity analysis and ≥ 0.25% in the study of QS conservation. The threshold for haplotype filtering was empirically selected based on results obtained by simultaneously sequencing known clones, as previously reported by our group^34,35^. Each haplotype was genotyped by a distance-based method, as previously reported^16,26^ (Supplementary Table S2). The method used to analyze the reference sequences is reported in a supplementary file (Supplementary method; Supplementary Table S3). As was observed by UPGMA (unweighted pair group method with arithmetic mean), this amplicon sufficed to differentiate the viral genotypes (Supplementary Figure S7), but it could not distinguish genotype subtypes because of its limited length.

### Quasispecies complexity

QS complexity was determined in patients with ≥ 10,000 reads (15/16 CI, 16/19 CHB, 14/16 HCC and 4/6 LC) by applying four parameters: Sn, G, Mf, and π^36^. The Sn and G are abundance indices that measure haplotype diversity based on the number of haplotypes and their relative frequencies. The functionality indices include the Mf, which measures the genetic diversity of the viral population with respect to the most prevalent haplotype, and the π index, which measures genetic diversity as the average number of mutations per site between each pair of haplotypes in the viral population^36^ (Supplementary Table S4).

### Quasispecies conservation

Sequence conservation was determined by calculating the information content of each position (both nt and aa) in a multiple alignment of all haplotypes obtained with NGS, followed by sliding window analysis, as previously described by our group^16^. To determine the intergroup variability in sequence conservation, the standard deviation of the mean overall information content was calculated for each group of patients.

### Mutation analysis

Each haplotype was aligned to a reference consensus sequence of the same genotype to detect aa changes. Genotype consensus was generated by aligning the region of interest (nt 1255–1611) extracted from full-length HBV genome sequences obtained from GenBank (13 sequences for genotype A, 23 genotype C, 17 genotype D, 8 genotype E, 10 genotype F, and 5 genotype H; Supplementary Table S3). The frequency of each aa change observed was calculated as the sum of the relative frequencies of each mutated haplotype in the patient’s QS population. The three-dimensional structures of HBx were predicted by I-Tasser^37^ using a validated HBx reference model^38^ as a custom-added modelling constraint.

The fold-stability change ΔΔg between wt and both mutation patterns were calculated by Strum^39^, with ΔΔg (WT-Mut) < 0 indicating reduced stability in presence of mutations.

### In vitro HBV expression in the presence of mutations

Mutations that were differentially represented between the patient groups were tested in vitro by HBV linear monomer transfection^40,41^.

The HBV monomer was obtained from the pCRII.HBV.ayw plasmid (kindly donated by Prof. Massimo Levrero and Dr. Laura Belloni), which contains a full-length HBV genotype D genome (subtype ayw). HBV monomer start/end nucleotide positions fall into L-HBsAg gene. The start encompassed nt 1 to nt 837, whereas the end included positions 2850–3182 of the same gene (positions are given in relation to the HBV ayw consensus sequence, NC_003977.2). Mutations were introduced by site-directed mutagenesis (QuikChange Lightening Site-Directed Mutagenesis kit, Agilent Technologies) using the manufacturer’s procedure, and mutated plasmids were isolated using the Plasmid Midi kit (QIAGEN) according to the manufacturer’s instructions. Linear HBV genomes, both wt and mutated, were obtained by digestion using EcoRI and PvuI, extracted from gels using the QIAquick Gel Extraction Kit (QIAGEN), and quantified with a Qubit fluorimeter (ThermoFisher).

HepG2-hNTCP cells were cultured with Dulbecco’s modified Eagle’s medium (DMEM) supplemented with 10% fetal bovine serum (FBS), penicillin (100 U/mL), streptomycin (100 μg/mL), Glutamax (2 mM), and Puromycin (5 μg/mL). To synchronize cells, they were treated with DMSO 2.5% at least 14 days before plating^42–44^. The day before transfection, cells were plated in 24-well plates at a density of 60,000 cells/mL in DMEM with 10% FBS (complete medium). The next day, cells were transfected with wt or mutated linear HBV monomer (250 ng/well) using the TransIT-X2 Dynamic Delivery System (Mirus). The pmaxFP-green plasmid (Amaxa Biosystem) was added at 1:10 to each well as transfection control. The medium was replaced the next day and changed every 2 days. Supernatants were collected at 5 days post-transfection. To remove residual linear DNA, supernatants were treated with DNAseI (Sigma, 1 mg/mL) in the presence of MgCl_2_ (25 mM), and the reaction was stopped after 1 h using EDTA (25 mM).

HBV DNA was quantified by real-time PCR (COBAS 6800, Roche Diagnostics) and hepatitis B core-related antigen (HBcrAg) was tested using a commercial chemiluminescent immunoassay (Lumipulse, Fujirebio) with a limit of detection of 2 logU/mL.

### Statistics

Intergroup differences for age, viremia, ALT, complexity indices, and aa changes were evaluated using the Kruskal–Wallis test and the Dunn posthoc test. Differences in QS conservation were evaluated by the Wilcoxon test, whereas differences in genotype distribution were assessed with ANOVA, followed by the Turkey test. The Kruskal–Wallis test and the Dunn posthoc test were used when comparing HBV DNA and HBcrAg titer in cell supernatants. *p* values were adjusted with the Bonferroni correction, and those < 0.05 were considered statistically significant. All tests were done with R language software (3.2.3)^45^.

## Congress presentation

This study was partially presented as poster presentation at the Liver Meeting 2018 of the American Association for the Study of Liver Disease in San Francisco, California, on 9 to 13 November, 2018, and at the International Liver Congress 2019 for the European for the European Association for the Study of the Liver 2019, in Vienna, Austria on the 10 to 14 April 2019.

## Supplementary Information

Supplementary Information.
