# Supplementary material for: Sophisticated viral quasispecies with a genotype-related pattern of mutations in the hepatitis B X gene of HBeAg-ve chronically infected patients
Source: Sci Rep. 2021 Feb 18;11:4215. doi: 10.1038/s41598-021-83762-4 (PMC7892877; doi:10.1038/s41598-021-83762-4)
Supplement: Supplementary file 1 — Supplementary Information. [file 41598_2021_83762_MOESM1_ESM.pdf]

Sophisticated viral quasispecies with a genotype-related pattern of mutations in the hepatitis B X gene of HBeAg-ve chronically infected patients

Cortese Maria Francesca[1,2,†,*], *González Carolina*[†,2], *Gregori Josep*[1,3], *Casillas Rosario*[1,2], *Carioti Luca*[4], *Guerrero-Murillo Mercedes*[5], *Riveiro Barciela Mar*[6,7], *Godoy Cristina*[2,6], *Sopena Sara*[1,2], *Yll Marçal*[1,2], *Quer Josep*[1,6], *Rando Ariadna*[2], *Lopez-Martinez Rosa*[2], *Pacin Ruiz Beatriz*[1], *García García Selene*[1], *Esteban-Mur Rafael*[6,7], *Tabernero David*[2,6], *Buti Maria*[6,7], *Rodríguez-Frías Francisco*[2,6]

Supplementary Files

Genotyping method

Reference sequences were selected from GenBank to represent all viral genotypes and subtypes. Reference sequences were deeply analyzed to eliminate duplicates and sequences that did not classify the viral genotypes well. Considering the limited length of the amplicon analyzed in this study, classification into viral subtypes was rejected. Reference sequences were later analyzed by a distance-based method and results were compared with those obtained by phylogenetic methods: neighbor joining and UPGMA (unweighted pair group method with arithmetic mean). There were no differences in the results obtained by these methods.

The patients' haplotypes were independently genotyped by the distance-based method and results were confirmed by applying a UPGMA tree.

Supplementary Figure S1. Comparison of QS complexity indices obtained using 2-round and 3-round PCR. Plasma from 2 CHB patients (panel A and B, respectively) were serially diluted up to e3, HBV DNA was extracted and amplified in the region of interest by 2-round (red square) or 3-round PCR (green triangle). The paired Wilcoxon test was implemented to compare indices obtained with the two PCR protocols (p= 0.5 and 0.1 for patient 1 and 2, respectively). Results were also confirmed by applying a Bland-Altman analysis (confidence interval = 0.95), obtaining similar p-value (p= 0.6 and 0.1 for patient 1 and 2).

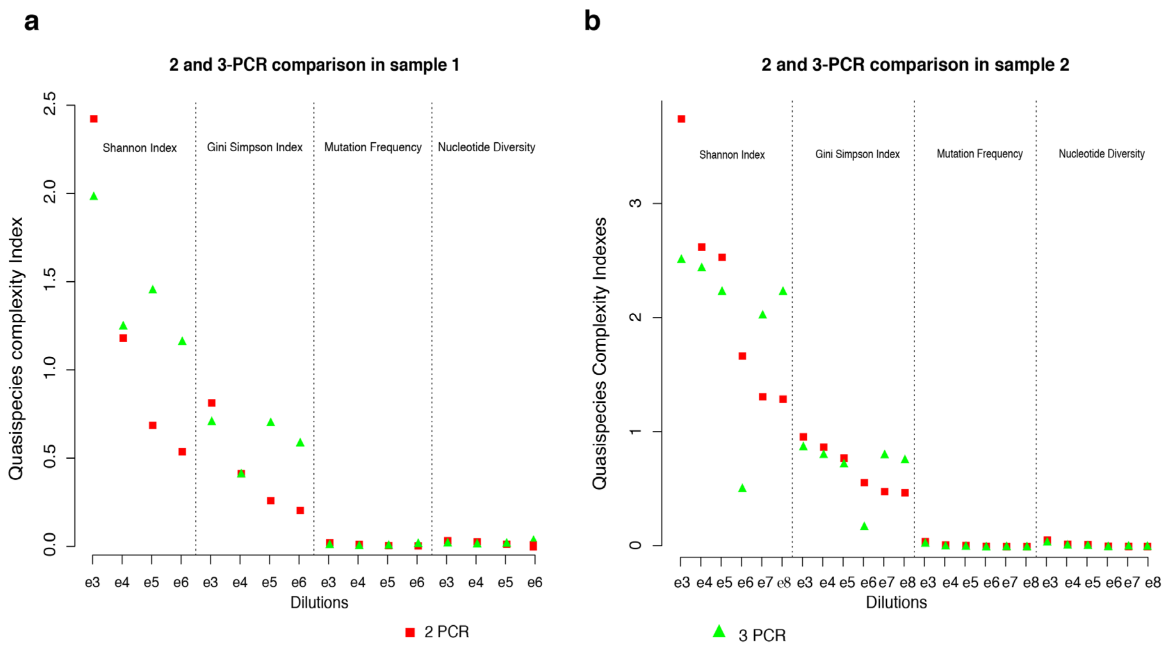

Supplementary Figure S2. QS conservation in multiple alignment of nucleotide sequences. a) The sliding window analysis is the result of the mean information content (bits) of the 25-nt long windows with a displacement between them of 1nt obtained by multiple alignment of all haplotypes (blue line) and taking into account their relative frequencies (red line). b) Sequence logos represent the information content of the most conserved regions detected in sliding window analysis. The height of each letter indicates the degree of conservation, from a minimum of 0 to a maximum of 2 bits (100% conservation). The positions of the hyperconserved regions are reported at the top of each logo.

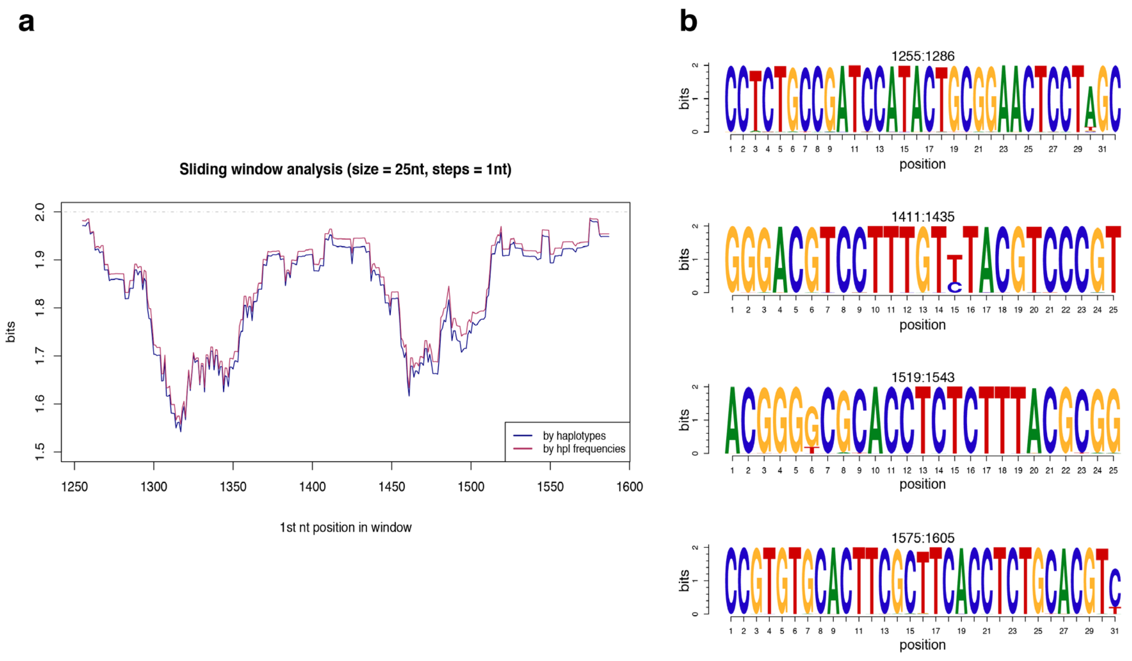

Supplementary Figure S3. QS conservation in multiple alignment of amino acid sequences. a. Sliding window analysis of the mean information content (bits) of the 10-aa long windows with a displacement between them of 1 aa obtained by multiple alignment of all aa haplotypes (blue line) and taking into account their relative frequencies (red line). b. Sequence logos represent the information content of the most conserved aa regions detected in sliding window analysis. 0 bits indicates minimum and 4 bits maximum conservation. The hyperconserved region positions are reported at the top of each logo.

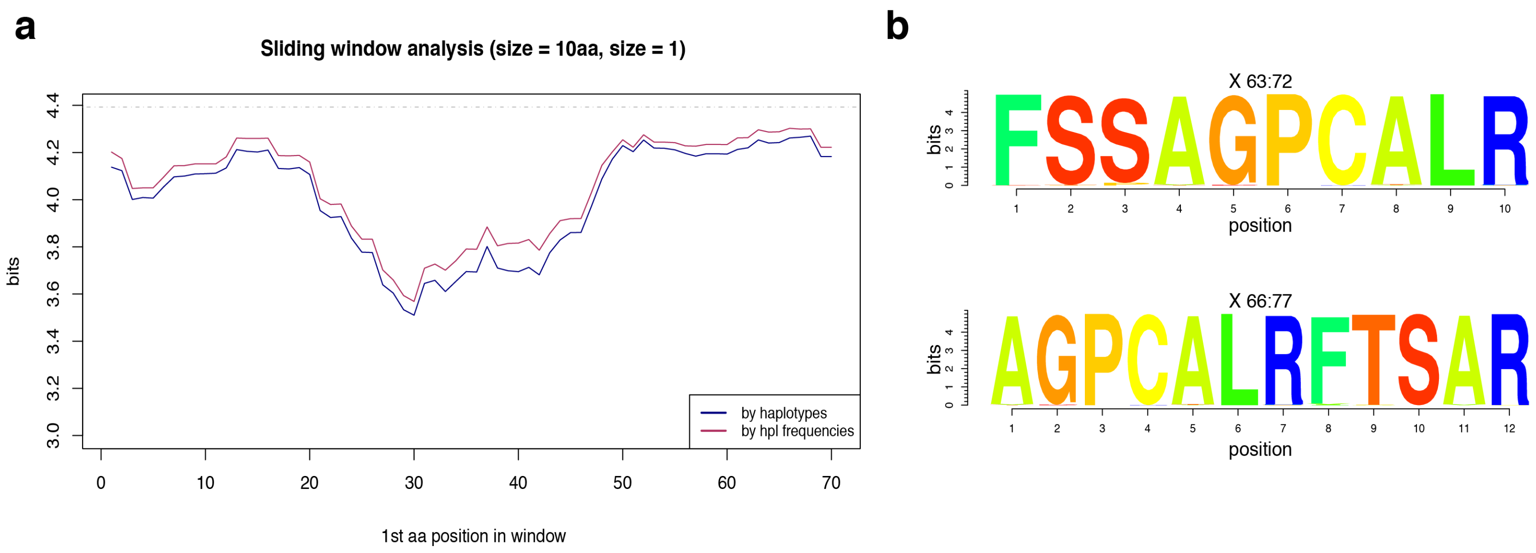

Supplementary Figure S4: Genotype-specific mutations. The Venn Diagram shows the number of mutations observed alone or in common in the various viral genotypes (red genotype D, green genotype E, blue genotype H, yellow genotype A, and grey genotype C). The number of genotype-specific mutations is indicated in red. The number of shared mutations is reported in black within the intersection space between the genotype ellipses. Genotype F is not represented because no mutations were detected. The A12S/P33S/P46S/T36G-D pattern is also included in the group of genotype D-specific mutations. The diagram was obtained through R language software (3.2.3)[45].

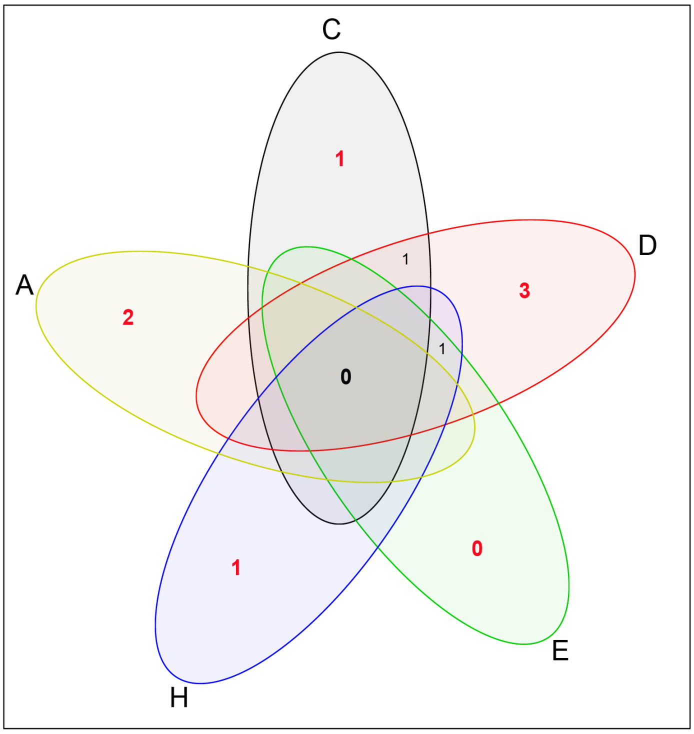

Supplementary Figure S5. HBx protein tridimensional structure. The figure shows the 3D structure of HBx protein as wt (a), in presence of the A12S/P33S/P46S/T36G pattern (b) and in presence of the A12S/P33S/P46S/T36D pattern (c). The pattern aa positions are highlight in green, and the aa changes are reported. Alpha-helices are shown in blue, beta-sheets in orange random coil in gray. The 3D structures were obtained by homology modeling of wt and mutated consensus in I-Tasser server.

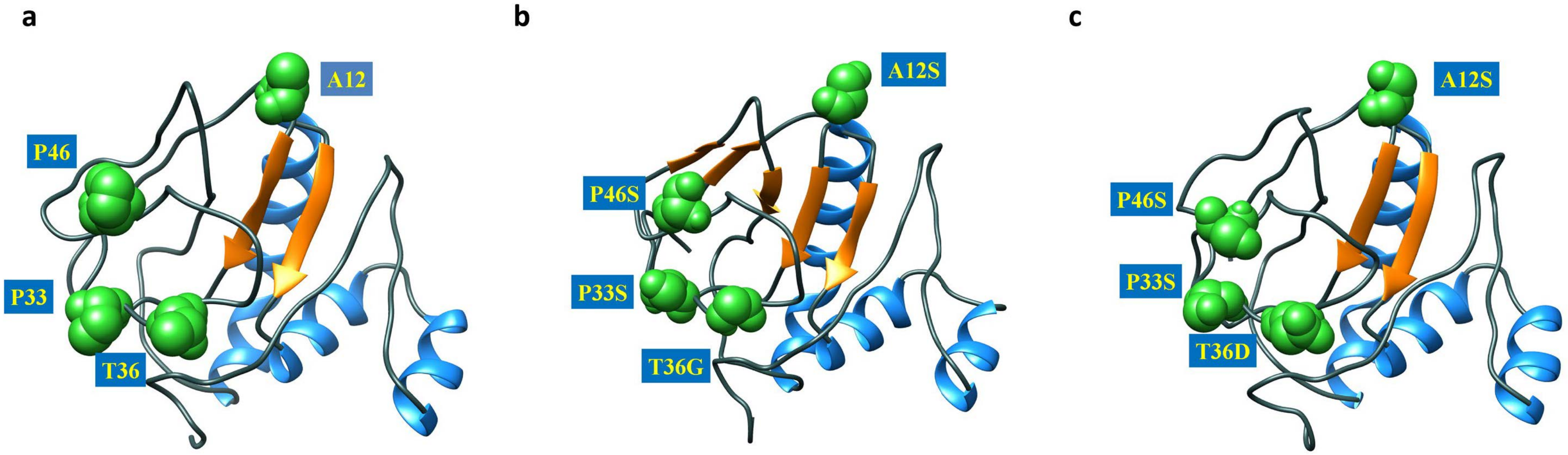

Supplementary Figure S6. HBcrAg release in the presence of the mutation pattern. The boxplot shows HBcrAg release in cell cultures 5 days after transfection with wt or mutated HBV linear monomer. Bonferroni-corrected p-values were calculated by applying the Kruskal-Wallis Test plus Dunn test and are represented by an asterisk (p = 0.04). Results are the median of three experiments performed in duplicate.

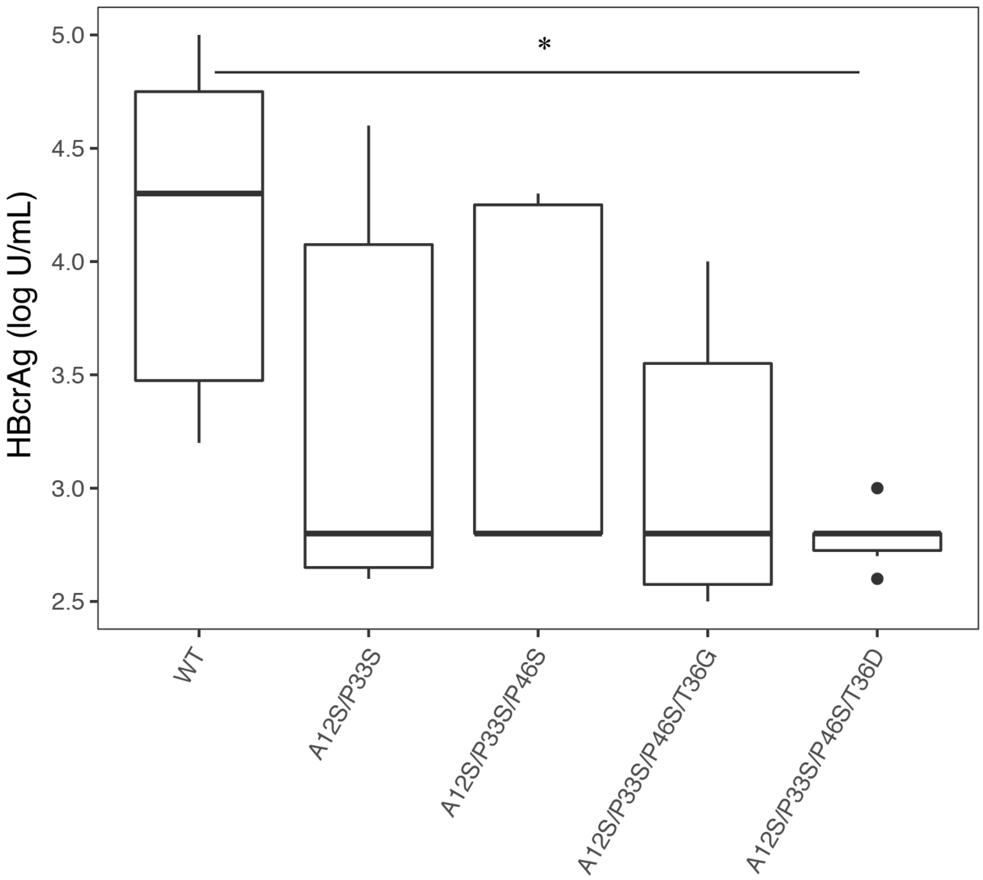

Supplementary Figure S7. 5'X amplicon discrimination of viral genotypes. The bootstrap shows the discrimination of viral genotypes based on the 5'X amplicon obtained from different GeneBank reference sequences. Viral genotypes are represented by different colors. The GeneBank reference number is reported.

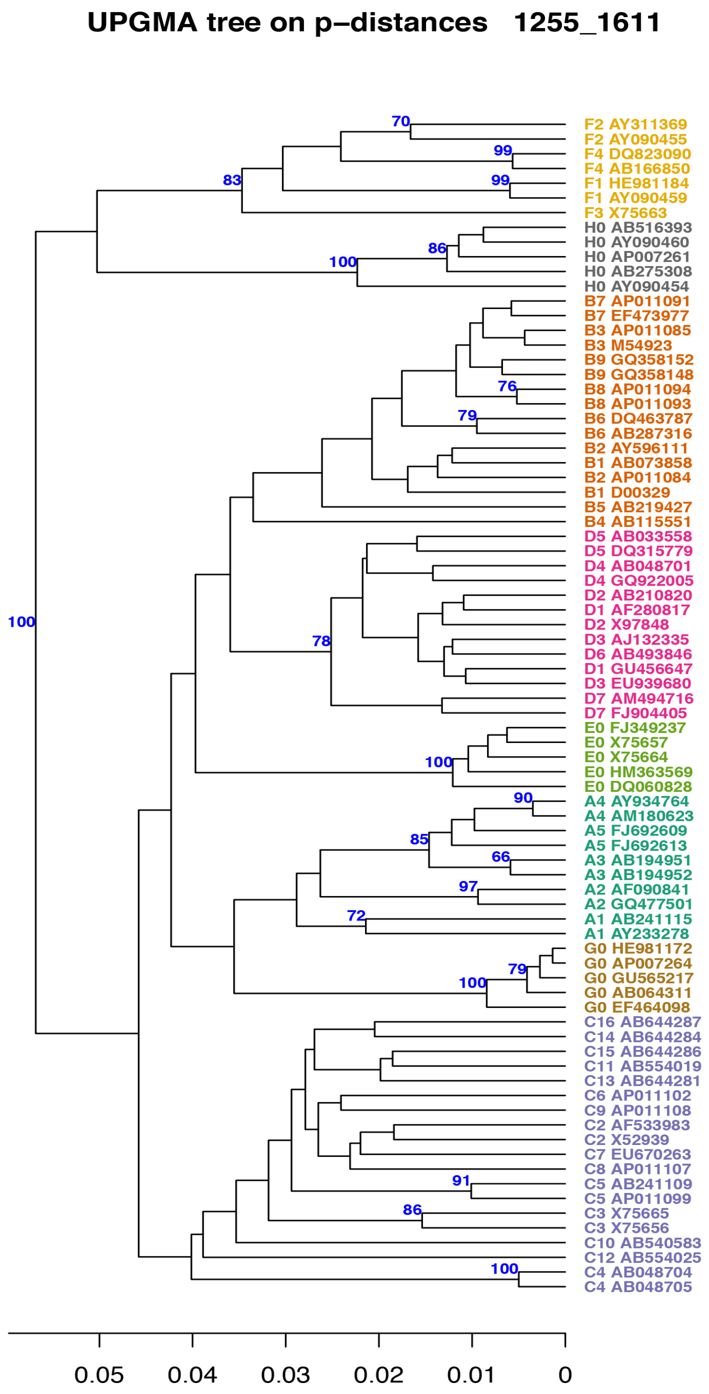

Supplementary Table S1. Biosample accession numbers for each sample analyzed

Clinical stage	**Biosample accession number**
CI	SAMN10077196; SAMN10077197; SAMN10077198; SAMN10077199; SAMN10077200; SAMN10077201; SAMN10077202; SAMN10077203; SAMN10077204; SAMN10077205; SAMN10077206; SAMN10077207; SAMN10077208; SAMN10077209; SAMN10077210; SAMN10077211
HCC	SAMN10077212; SAMN10077213; SAMN10077214; SAMN10077215; SAMN10077216; SAMN10077217; SAMN10077218; SAMN10077219; SAMN10077220; SAMN10077221; SAMN10077222; SAMN10077223; SAMN10077224; SAMN10077225; SAMN10077226; SAMN10077227
LC	SAMN10077228; SAMN10077229; SAMN10077230; SAMN10077231; SAMN10077232; SAMN10077233
CHB	SAMN10077234; SAMN10077235; SAMN10077236; SAMN10077237; SAMN10077238; SAMN10077239; SAMN10077240; SAMN10077241; SAMN10077242; SAMN10077243; SAMN10077244; SAMN10077245; SAMN10077246; SAMN10077248; SAMN13948585; SAMN13948586; SAMN13948587; SAMN13948588; SAMN14411169

CI: Chronic infected; HCC: Hepatocellular carcinoma; LC: Liver cirrhosis, CHB: Chronic hepatitis B.

Supplementary Table S2. Genotype distribution of haplotypes per patient

Patient	Group	A %	B %	C %	D %	E %	F %	H %
1	IC	0	0	0	98	0.9	0.7	0
2	IC	7.7	0	1.1	91.2	0	0	0
3	IC	1.0	0	5.2	93.8	0	0	0
4	IC	1.8	0	0	98.2	0	0	0
5	IC	0	0	0	99.2	0	0	0.8
6	IC	0	0	0	100	0	0	0
7	IC	0	0	18.1	81.9	0	0	0
8	IC	0	0	0	16.3	0	83.7	0
9	IC	0	0	47.0	53.0	0	0	0
10	IC	0	0	0.3	93.5	0	0	6.2
11	IC	0.5	0	48.7	50	0	0.4	0
12	IC	29.0	0	4.0	67	0	0	0
13	IC	0.7	0	55.1	44.2	0	0	0
14	IC	0	0	0	45.7	0	54.4	0
15	IC	0.4	0	17.0	82.7	0	0	0
16	IC	0	0	10.0	90.0	0	0	0
17	HCC	96.7	0	2.5	0.9	0	0	0
18	HCC	0	0	93.4	6.6	0	0	0
19	HCC	0	0	100	0	0	0	0
20	HCC	0	0	100	0	0	0	0
21	HCC	0	0	0	100	0	0	0
22	HCC	100	0	0	0	0	0	0
23	HCC	1.0	0	8.8	90.3	0	0	0
24	HCC	100	0	0	0	0	0	0
25	HCC	89.4	0	2.8	7.8	0	0	0
26	HCC	100	0	0	0	0	0	0
27	HCC	17.4	0	0	82.6	0	0	0
28	HCC	0	0	0	91.6	0	0	8.4
29	HCC	0	0	0	100	0	0	0
30	HCC	100	0	0	0	0	0	0
31	HCC	0	0	0	100	0	0	0
32	HCC	100	0	0	0	0	0	0
33	LC	0	0	13.8	85.8	0.4	0	0
34	LC	0.9	0	8	39.5	0	0	51.6
35	LC	2.1	0	9.3	57.5	0	31.1	0
36	LC	0	0	0	100	0	0	0
37	LC	6.4	0	41.2	52	0	0	0
38	LC	0	0	12.3	87.7	0	0	0
39	CHB	0	0	0	0	100	0	0
40	CHB	7.5	0	2.1	7.44	42.0	0	40.9
41	CHB	100	0	0	0	0	0	0
42	CHB	0	0	98.2	1.78	0	0	0
43	CHB	100	0	0	0	0	0	0
44	CHB	0	0	1.9	4.04	94.1	0	0
45	CHB	0	0	0	100	0	0	0
46	CHB	0	0	0	0	0	0	100
47	CHB	0	0	2.2	12.6	85.2	0	0
48	CHB	0	0	0	0	0	0	100
49	CHB	0	0	0	0.4	0	99.3	0
50	CHB	56.4	0	4.1	39.54	0	0	0
51	CHB	0	0	0	100	0	0	0
53	CHB	0	0	0	100	0	0	0
54	CHB	0.4	0	2.3	97.3	0	0	0
55	CHB	40.2	0	2.5	38.7	0	0	18.6
56	CHB	0.4	0	2.1	97.5	0	0	0
57	CHB	14.9	0	3.3	81.7	0	0	0
58	CHB	0	0	19.3	80.72	0	0	0

Supplementary Table S3. NCBI GenBank accession numbers of 102 reference sequences used in HBV genotyping. The consensus reference sequence used for the analysis of amino acid changes was obtained by aligning these sequences per each genotype.

HBV genotype (N sequences)	GenBank accession numbers
A (13)	AY233278, AB241115, GQ477501, AF090841, AF090839, AB194952, AB194951, AM180623, AY934764, FJ692609, FJ692613, GQ331047, GQ331048
B (20)	AB073858, AB362933, D00329, AY596111, AP011084, GQ924653, M54923, AP011085, AB073835, AB115551, AB219427, AP011086, AB287316, DQ463787, EF473977, AP011091, AP011093, AP011094, GQ358148, GQ358152
C (23)	AB112066, X52939, AF533983, AB033553, X75656, X75665, AB048705, AB048704, AP011099, AB241109, AP011102, AP011103, EU670263, AP011107, AP011104, AP011108, AB540583, AB554019, AB554025, AB644281, AB644284, AB644286, AB644287
D (17)	AB555496, GU456647, AB104712, AF280817, AB210820, X97848, Z35716, EU939680, AY233291, AJ132335, GQ922005, AB048701, DQ315779, AB033558, AB493846, FJ904405, AM494716
E (8)	X75664, X75657, FJ349237, AM494694, HM363569, FJ349226, DQ060828, JQ000008
F (10)	AY090459, HQ378247, HE981184, AY090455, AY311369, X69798, X75663, AB036911, AB166850, DQ823090
G (6)	EF464098, HE981172, HE981176, AP007264, GU565217, AB064311
H (5)	AP007261, AB275308, AY090460, AY090454, AB516393

Supplementary Table S4. Definitions of the quasispecies complexity indices

**Indices**	**Definition**	**Formula**	
**Shannon Entropy Index (Sn)‡**	The grade of uncertainty in predicting sequences identity when taken on random. Quasispecies population with more haplotypes at low frequencies will present higher value of Sn.	$-\sum_{i=1}^{H} p_i \log(p_i)/\log(H)$	*pi* = frequency of haplotype *i* in the quasispecies population<br>*H* = number of haplotypes in the quasispecies population
**Gini Simpson Index (G)‡**	The probability that two randomly taken genomes belong to different haplotypes. G will have a higher value in quasispecies with a larger number of haplotypes at low frequency,	$1-\sum_{i=1}^{H} p_i^2$	*pi* = frequency of haplotype *i* in quasispecies population
**Mutation Frequency (Mf)§**	The fraction of mutated residues in the multiple alignments with respect to the dominant haplotype.	$\frac{1}{H}\sum_{i01}^{H} d_{1i}$	d = proportion of mutations related to the master sequence
**Nucleotide Diversity (π)§**	The average number of substitutions between pairs of haplotypes in the multiple alignments.	$\sum_{i=1}^{H}\sum_{j=1}^{H} p_i d_{ij} p_j$	*pi* = frequency of haplotype *i* in the viral quasispecies<br>*pj* = frequency of haplotype *j* in the viral quasispecies<br>dij = Hamming distance (number of mutations that differ haplotype *i* and *j*.

‡ Abundance indices = take into account haplotypes in the QS and their abundance (in terms of relative frequency)
§ Functional indices = based on the genetic differences between haplotypes (known as genetic distance)
